# Atom mapping with constraint programming

**DOI:** 10.1186/s13015-014-0023-3

**Published:** 2014-11-29

**Authors:** Martin Mann, Feras Nahar, Norah Schnorr, Rolf Backofen, Peter F Stadler, Christoph Flamm

**Affiliations:** Bioinformatics Group, Department of Computer Science, University of Freiburg, Georges-Koehler-Allee 106, Freiburg, 79110 Germany; Centre for Biological Signalling Studies (BIOSS), University of Freiburg, Freiburg, Germany; Centre for Biological Systems Analysis (ZBSA), Cluster of Excellence, University of Freiburg, Habsburgerstr. 49, Freiburg, 79104 Germany; Center for non-coding RNA in Technology and Health, University of Copenhagen, Grønnegårdsvej 3, Frederiksberg, 1870 Denmark; Institute for Theoretical Chemistry, University of Vienna, Währingerstrasse 17, Vienna, 1090 Austria; Bioinformatics Group, Department of Computer Science, and Interdisciplinary Center for Bioinformatics, University of Leipzig, Härtelstraße 16-18, Leipzig, 04107 Germany; Max Planck Institute for Mathematics in the Sciences, Inselstraße 22, Leipzig, 04107 Germany; Fraunhofer Institute for Cell Therapy and Immunology, Perlickstraße 1, Leipzig, 04103 Germany; Santa Fe Institute, 1399 Hyde Park Rd., Santa Fe, 87501 NM USA

**Keywords:** Atom-atom mapping, Constraint programming, Chemical reaction, Imaginary transition state

## Abstract

Chemical reactions are rearrangements of chemical bonds. Each atom in an educt molecule thus appears again in a specific position of one of the reaction products. This bijection between educt and product atoms is not reported by chemical reaction databases, however, so that the “Atom Mapping Problem” of finding this bijection is left as an important computational task for many practical applications in computational chemistry and systems biology. Elementary chemical reactions feature a cyclic imaginary transition state (ITS) that imposes additional restrictions on the bijection between educt and product atoms that are not taken into account by previous approaches. We demonstrate that Constraint Programming is well-suited to solving the Atom Mapping Problem in this setting. The performance of our approach is evaluated for a manually curated subset of chemical reactions from the KEGG database featuring various ITS cycle layouts and reaction mechanisms.

**Electronic supplementary material:**

The online version of this article (doi:10.1186/s13015-014-0023-3) contains supplementary material, which is available to authorized users.

## Background

A chemical reaction describes the transformation of a set of educt molecules into a set of products. In this process, chemical bonds are re-arranged, while the atom types remain unchanged. Thus, there is a one-to-one correspondence, the so-called *atom map* (or atom-atom mapping), between the atoms of educts and products. Atom maps convey the complete information necessary to disentangle the mechanism, i.e., the bond re-arrangement, of a chemical reaction because they unambiguously identify the bonds that differ between educt and product molecules. The changing parts of the molecules are described by a so called imaginary transition state (ITS) [1,2] that allows, for instance, a classification of chemical reactions [3-5]. Atom maps are a necessary requisite for computational studies of an organism’s metabolism. For instance, they allow for consistency checks within metabolic pathway analyses [6] and play a key role in the global analysis of metabolic networks [7,8]. Practical applications include, for example, the tracing or design of the metabolic break down of a candidate drug, which constitutes an important issue in drug design studies [9].

Only the product and educt molecules involved in a chemical reaction are directly observable. The atom map therefore often remains unknown and has to be inferred from partial knowledge. Experimental evidence may be available from isotope labeling experiments. Here, special isotopes, i.e. atoms with special variations, are introduced into educt molecules that can then be identified in product molecules by means of spectroscopy techniques [10]. Such data, however, is not available for most reactions. The complete experimental determination of an atom map is in general a complex and tedious endeavor. Reaction databases, such as KEGG, therefore do not generally provide atom maps. The computational construction of atom maps is therefore an important practical problem in chemoinformatics [11].

**Figure 1:**
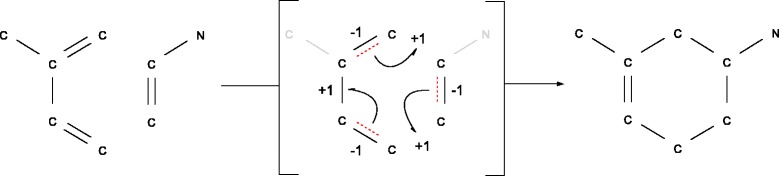
**Diels-Alder reaction.** Example of a Diels-Alder reaction omitting hydrogen atoms. The imaginary transition state (ITS) is an alternating cycle defined by the bonds that are broken (dotted) and the bonds that are newly formed.

Several computational approaches for this problem have been developed over the last three decades (for a recent review see [12]). The educts and products are described as two not necessarily connected labeled graphs *I* and *O*, respectively. Vertex labels define atom types, while edge labels indicate bond types. The atom map is then determined as the solution of a combinatorial optimization problem resulting in a bijective mapping of all vertices of the educt molecule graph to corresponding vertices in the product molecule graphs. An illustration of a Diels-Alder reaction is given in Figure 1.

The most common formulations are variants of the maximum common subgraph (isomorphism) problem [13]. Already the earliest approaches analyzed the adjacency information within educts and products [14,15]. The Principle of Minimal Chemical Distance, which is equivalent to minimizing an edge edit distance, was invoked in [16], using a branch and bound approach to solve the corresponding combinatorial optimization problem. Maximum Common Edge Subgraph (MCES) algorithms search for isomorphic subgraphs of the educt/product graphs with maximum number of edges [4,17-20], an NP-hard problem. Furthermore, the use of specialized energetic [21,22] or weighting [23] criteria allows for the identification of the static parts of the reaction and, subsequently, of the atom mapping. A detailed investigation of the MCES from an Integer Linear Programming (ILP) perspective can be found in [24].

Akutsu [25] showed that the MCES approach fails for certain reactions. As an alternative, the Maximum Common Induced Subgraph (MCIS) problem was proposed as a remedy. This problem is also NP complete. Approximation results can be found in [26]. Algorithms for the MCIS iteratively decompose the molecules until only isomorphic sub-graphs remain [7,25,27,28]. Recently, an ILP approach incorporating stereochemistry was presented [29].

Neither the solutions of the MCES nor the MCIS necessarily describe the true atom map. Indeed, both optimality criteria are artificial and can not be derived from basic principles of chemical reactions. In fact, it is not hard to construct counter-examples, i.e., chemical reactions whose true atom maps are neither identified by MCES nor by MCIS. The re-organization of chemical bonds in a chemical reaction is far from arbitrary but follows strict rules that are codified e.g. in the theory of imaginary transition states (ITS) [1,2]. The ITS encodes the redistribution of bond electrons that occurs along a chemical reaction. Bond electrons define the atom-connecting chemical bonds and their according bond orders. Their redistribution is expressed in terms of the deletion or formation of bonds as well as changes of the oxidation state of atoms, the latter resulting from non-bound electrons that are freed from or integrated into bonds. The ITS can be used to cluster, classify, and annotate chemical reactions [1,2,30]. These studies revealed that only a limited number of ITS “layouts” are found among single step reactions and that these layouts represent a cyclic electron redistribution pattern usually involving less than 10 atoms [30]. In a most basic case, an elementary reaction, the broken and newly formed bonds form an alternating cycle (see Figure 1) covering a limited even number of atoms [31], usually less than 8 [2]. In the case of homovalent reactions, i.e., those in which the number of non-bound electron pairs of all atoms (defining their oxidation state) remains unchanged, this cycle is elementary. That is, the transition state is a single, connected even cycle, along which bond orders change by ±1 [30]. This property imposes an additional, strong condition of the atom maps that is not captured by the optimization approaches outlined in the previous paragraphs. Here, we explicitly include it into the specification of the combinatorial problem.

A *chemically correct* atom map is a bijective map between the vertices of the educt and product graphs such that:

1. The map preserves atom types
2. The total bond orders (including lone electron pairs) are preserved. Each broken bond thus must be compensated by a newly formed bond or a change in the oxidation number of an atom.
3. The broken and newly formed bonds constitute a chemically reasonable imaginary transition state (ITS) following [30]. In the case of elementary chemical reactions, the transition state is an alternating cycle.

A formal definition of the combinatorial problem will be given in the following section. While cyclic transition states are very common, more “complex transition states” appear in non-elementary reactions, i.e., compositions of elementary reactions. Furthermore, even in elementary reactions, it is not true that a shortest ITS cycle is necessarily chemically correct. Empirically, transition states are most frequently six-membered cycles, while cycles of length 4 or 8 are less abundant [1,2,31,32]. As a consequence, we will consider several variants of the chemical reaction mapping problem:

1. **Decision problem**: Is there an atom map with cyclic ITS? Of course one may restrict the question to ITS cycles of length *k*.
2. **Optimization problem:** Find the minimal length *k* of an ITS cycle that enables an atom map.
3. **Enumeration problem:** Find all atom maps with cyclic ITS (of length *k*).

Given a straightforward encoding of molecular graphs in terms of vertex indices, atom labels, and adjacency information, the atom mapping problem is naturally open to be treated as a constraint satisfaction problem with finite integer domains. This approach is particularly appealing when additional information on the ITS, e.g. its size or atoms involved in the ITS, are known. The theory and model of such a constraint-based atom mapping approach was introduced by us in [33]. This manuscript is an extended version of [33]. Here, we provide a more detailed description of the formalisms and evaluate the performance of the approach on a large reaction data set. The latter was manually curated and compiled to enable a validation of the computational predictions.

## Constraint programming formulation of the atom mapping problem

We focus on the identification of the cyclic ITS. Once the ITS has been identified the overall atom mapping is easily derived. We formulate separate constraint satisfaction problems for different ITS layouts and cycle lengths. A fast graph matching approach is used subsequently to extend each ITS to a global atom mapping. In this section we follow closely [33]. We first formally define the problem, which is followed by a description of our constraint programming approach for identifying the cyclic ITS. Finally we discuss how to extend an ITS candidate to a complete atom mapping for the chemical reaction.

### Problem definition

Both educts and products of a chemical reaction are each represented by a single, not necessarily connected, undirected graph defined by a set of vertices *V* and a set of edges *E*⊆{ {*v*,*v*^′^} | *v*,*v*^′^∈*V*}. The educt (input) graph is denoted by *I*=(*V*_*I*_,*E*_*I*_) and the product (output) graph by *O*=(*V*_*O*_,*E*_*O*_). Here, each molecule corresponds to a connected component. Vertices represent atoms and are labeled with the respective atom type accessible via the function *l*(*v*∈*V*_*I*_∪*V*_*O*_). The principle of mass conservation implies |*V*_*I*_|=|*V*_*O*_|, i.e. no atom can dissolve or appear during a reaction. Edges encode covalent chemical bonds between atoms. For the CSP formulation we label each edge {*x*,*y*}∈*E*_*I*_∪*E*_*O*_ with the number of shared electron pairs, i.e., its bond order: single, double or triple bonds are represented by a single edge with labels 1, 2, or 3, respectively. Note, this molecule representation ignores stereochemistry, i.e. there is no differentiation between the optimal isomers of chiral molecules. Non-bonding electron pairs of an atom, which define its oxidation state, are represented by self loop edges labeled with the according number of unbound pairs.

We use an adjacency matrix  to encode the edge labels of the educt graph (and a corresponding matrix  for the products). The matrix elements $\mathcal {I}_{v,v^{\prime }}$ denote the number of shared bond electron pairs for the edge between the atoms *v* and *v*^′^ in the educt graph *I*. In practice $\mathcal {I}_{v,v'}\in \{0,1,2,3\}$, where 0 means no electrons are shared. Non-bonding electron pairs (loops) are represented by the diagonal entries $\mathcal {I}_{v,v}$ and $\mathcal {O}_{v,v}$. Now consider a bijective function *m*:*V*_*I*_→*V*_*O*_ mapping the vertices of *I* onto the vertices of *O*. We can use the mapping inversion *m*^−1^ to make the indexing of  compatible with . This is defined by $\mathcal {I}\circ m$, which is the matrix with *x*,*y* entries $=\mathcal {I}_{m^{-1}(x),m^{-1}(y)}$, i.e. with rows and columns indexed by *V*_*O*_. Based on that, we define the *reaction matrix*$\mathcal {R}^{m} = \mathcal {O}-(\mathcal {I}\circ m)$ as the elementwise matrix subtraction of  and the reindexed , which encodes the charge and bond electron differences between educts and products.

**Definition.** An *atom mapping* or *atom map* is a bijection *m*:*V*_*I*_→*V*_*O*_ such that

1. $\forall _{x\in V_{I}} : l(x) = l(m(x))$ (preservation of atom types)
2. $\mathcal {R}^{m} \overrightarrow {1}=\overrightarrow {0}$ (preservation of bond electrons for each atom)

The reaction matrix $\mathcal {R}^{m}$ encodes the imaginary transition state (ITS) [1,2]. This definition of *m* is a slightly more formal version of the Dugundji-Ugi theory [14]. Our notation emphasizes the central role of the (not necessarily unique) bijection *m*. Since we consider *I* and *O* as given fixed input, the atom mapping *m* uniquely determines $\mathcal {R}^{m}$. The triple (*m*,*I*,*O*), furthermore, completely defines the chemical reaction. It therefore makes sense to associate properties of the chemical reaction directly with the atom map *m*.

Equivalently, the ITS can be represented as a graph *R*=(*V*_*R*_,*E*_*R*_) so that *E*_*R*_ consists of the “changing” edges that lose or gain bond electrons during the reaction, i.e. $\mathcal {I}_{v,v'} \neq \mathcal {O}_{m(v),v(v^{\prime })} \leftrightarrow \mathcal {R}^{m}_{v,v^{\prime }} \neq 0$. The set of atom vertices *V*_*R*_⊆*V*_*O*_ covers all vertices with at least one adjacent edge in *E*_*R*_. Each edge {*v*,*v*^′^}∈*E*_*R*_ is labeled by the electron change $\mathcal {R}^{m}_{v,v'} \neq 0$, i.e. its change in bond order. See the following example adjacency matrices  and  for the reaction given in Figure 1.



The vertices *v*_*i*_∈*V*_*I*_ and $v^{\prime }_{j} \in V_{O}$ are numbered in top-down-left-right order of their appearance in Figure 1. The atom mapping $m(v_{i})= v^{\prime }_{i}$ defines $\mathcal {R}^{m}$ and thus the ITS graph *R* covers only vertices $v^{\prime }_{2}$ to $v^{\prime }_{7}$ since $v^{\prime }_{1}$ and $v^{\prime }_{8}$ do not show any bond electron changes.

It is important to note that the existence of an atom mapping *m* as defined above does not necessarily imply that $\mathcal {R}^{m}$ is a chemically plausible ITS.

We say that two edges {*v*,*v*^′^},{*v*^′^,*v*^′′^}∈*E*_*R*_ in *R* are *alternating* if $\mathcal {R}^{m}_{v,v^{\prime }}\neq 0$ and $\mathcal {R}^{m}_{v,v'}+\mathcal {R}^{m}_{v^{\prime },v^{\prime \prime }} = 0$. A *simple cycle* in *R* of size *k*>2 is given by the vertex sequence (*v*_1_,*v*_2_,…,*v*_*k*_,*v*_1_) with *v*_*i*_∈*V*_*R*_, {*v*_*i*_,*v*_*i*+1_}∈*E*_*R*_, {*v*_*k*_,*v*_1_}∈*E*_*R*_, and ∀*i*<*j*≤*k*:*v*_*i*_≠*v*_*j*_. Such a simple cycle is called alternating if all successive edges as well as the cycle closure {*v*_2_,*v*_1_},{*v*_1_,*v*_*k*_} are alternating.

**Definition.** An atom map *m* is *homovalent* if $\mathcal {R}^{m}_{v,v}=0$ for all *v*∈*V*_*R*_. A homovalent reaction is *elementary* if its ITS *R* is a simple alternating cycle. Thus $\mathcal {R}^{m}_{v,v^{\prime }}\in \{-k,0,+k\}$ with an absolute bond order change of $k\in \mathbb {N}^{+}$ holds for all elementary homovalent reactions.

In the following we outline a novel algorithm for finding atom maps for a given ITS graph *R* that is guaranteed to retrieve all possible mappings given the educt and product graphs  and , respectively. To simplify the presentation, first only elementary homovalent reactions with a bond order change of ±1 are considered. Generalizations are discussed in Section ‘Application and evaluation’.

### Constraint programming approach

The central problem to find an elementary homovalent atom mapping is to identify the alternating cycle defining the ITS *R* given the adjacency information of the educts  and products . This can be done via solving the Constraint Satisfaction Problem (CSP) as presented below. Note, due to the alternating edge condition within the ITS, we have to consider cycles with an even number of atoms only. In practice, the ITS of elementary homovalent reactions involves |*V*_*R*_|=4, 6, or 8 atoms [31].

#### Basic CSP formulation

In the following, we will present a first basic CSP for an ITS of size *k*=|*V*_*R*_| that we already introduced in [33]. It is given by the triple (*X*,*D*,*C*) defining the set of variables *X*, according set of domains *D*, and the set of constraints *C*. A solution is an assignment *A* that maps each variable *X*_*i*_∈*X* to a value *A*_*i*_∈*D*_*i*_ from its domain such that all constraints in *C* are fulfilled.

We construct an explicit encoding of the ITS atom mapping using *k* variables representing the cycle in *I* and another set for the *k* mapped vertices in *O*, i.e., $X = \left \{{X^{I}_{1}},\ldots,{X^{I}_{k}}\right \} \cup \left \{{X^{O}_{1}},\ldots,{X^{O}_{k}}\right \}$ with domains ${D^{I}_{i}}=V_{I}$ and ${D^{O}_{i}}=V_{O}$. Note, we do *not* directly encode the overall atom mapping problem but the identification of the two ITS subgraphs in the educts and products. Given this information, the overall atom mapping is easily identified as explained later.

To find a bijective mapping we have to ensure $\forall {i\neq j}:{X^{I}_{i}} \neq {X^{I}_{j}}$ and $\forall {i\neq j}:{X^{O}_{i}} \neq {X^{O}_{j}}$, i.e., a distinct assignment of all variables. To enforce atom label preservation we require consistency of labels for ${X^{I}_{i}}$ and ${X^{O}_{i}}$, i.e., an assignment *A* fulfills $l\left ({A^{I}_{i}}\right) = l\left ({A^{O}_{i}}\right)$.

Analogously, homovalence is represented by $\left (\mathcal {I}_{{A^{I}_{i}},{A^{I}_{i}}}\;-\mathcal {O}_{{A^{O}_{i}},{A^{O}_{i}}}\right) = 0$. Due to the alternating bond condition, each atom can lose or gain at most one edge during a reaction. Thus, we can further constrain the assignment with $|\text {degree}\left ({A^{I}_{i}}\right)-\text {degree}\left ({A^{O}_{i}}\right)| \leq 1$; here degree(*v*) denotes the out-degree of vertex *v*.

Finally, we have to encode the alternating cycle structure of the ITS in the mapping, i.e., for the sequence of bonds with indices 1-2-..-*k*-1. For all index pairs within the cycle (*i*,*j*) we therefore require pairs with even index *i* to correspond to the formation of a bond, i.e., we enforce $\left (\mathcal {O}_{{A^{O}_{i}},{A^{O}_{j}}}-\mathcal {I}_{{A^{I}_{i}},{A^{I}_{j}}}\right)=1$, while all odd indices *i* are bond breaking $\left (\mathcal {O}_{{A^{O}_{i}},{A^{O}_{j}}}-\mathcal {I}_{{A^{I}_{i}},{A^{I}_{j}}}\right)=-1$ accordingly.

**Figure 2:**
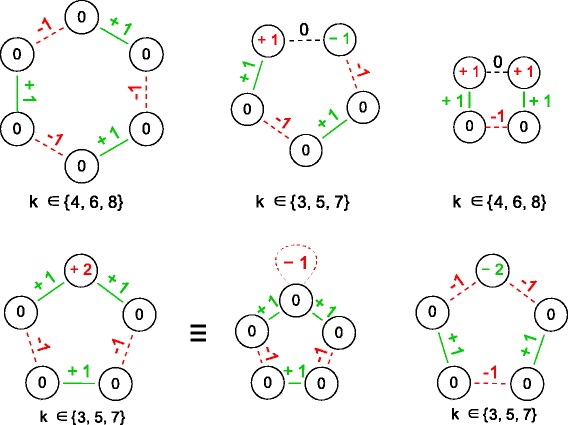
**Supported ITS layouts.** (top) ITS layouts found within the elementary reaction data set from [34]. The number within the vertices corresponds to atomic oxidation state changes, broken bonds are dotted given a negative bond label while formed bonds show positive numbers. (left) Homovalent elementary reactions result in even sized cycles with no oxidation state changes at the atoms (see Figure 1). (middle) Odd cycles with two oppositely charged atoms separated by a non-changing pseudo bond (dashed edge labeled 0 see Figure 5). (right) Similar layout involving two equivalent oxidation state changes. Note, the inverse layout was also found and used. (bottom) Additionally supported ITS layouts for ambivalent elementary reactions involving non bonding electrons. These result in odd sized cycles and oxidation state changes of one atom. Note that this situation is equivalent to a non-elementary cycle with alternating bond labeling (middle).

The homovalent ITS layout is rotation symmetric in itself (see Figure 2). To partially counter this, we introduce order constraints on the input variables: $\left (\forall {i>1}: {X^{I}_{1}} < {X^{I}_{i}}\right)$ using e.g. an index order on the vertices. This ties the smallest cycle vertex to the first variable ${X^{I}_{1}}$ and prevents the rotation-symmetric assignments of the input variables. Note, since we constrain the bond (1,2) to be a bond breaking $\left (\mathcal {O}_{{A^{O}_{1}},{A^{O}_{2}}}-\mathcal {I}_{{A^{I}_{1}},{A^{I}_{2}}}=-1\right)$, the direction of the cycle is fixed and all direction symmetries are excluded as well.

As we will show in the evaluation (Section ‘Application and evaluation’), the basic CSP will produce many ITS candidates that do not extend to an atom mapping over the whole educt and product graphs. Therefore, we introduce an extended version of this CSP that incorporates further constraints derived from the input.

#### Extended CSP formulation

**Figure 3:**
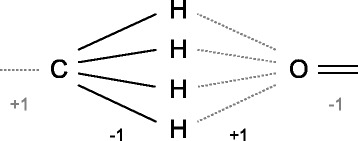
**Hydrogen symmetry problem.** Symmetries resulting from interchangeable hydrogens. The figure presents three successive atom assignments within an ITS mapping. Bonds present in *I* are given in black, bonds to be formed to derive *O* are dotted and gray. The ITS describes the loss of a hydrogen for the carbon (bond order decrease) and the bond formation between the decoupled hydrogen with the oxygen next in the ITS. It becomes clear that all 4 hydrogens are not distinguishable, which results in 4 possible symmetric ITS mappings.

Investigating the given educt and product graph, we can exclude a large set of symmetric solutions that arise due to an exchange of hydrogens. The latter can form at most one single bond to other atoms. Thus, if a hydrogen participates in the ITS, its adjacent atom will do as well (since the bond is to be broken in the ITS). Most adjacent atoms are non-hydrogens, e.g. carbon atoms, that can have multiple adjacent hydrogens. Since there is exactly one bond breaking and formation for each ITS atom, only one such adjacent hydrogen will be part of the ITS. This results in a combinatorial explosion due to the symmetries of adjacent hydrogen atoms. The latter results from the missing chirality information within the molecular graph encoding (see Problem definition). An example is given in Figure 3. To break this type of symmetry, we select for each non-hydrogen one adjacent “master” hydrogen (e.g. the one with lowest vertex index) and remove all other sibling hydrogens from the domains, both for educt and product variables *X*^*I*^ and *X*^*O*^, respectively. The hydrogen vertices to remove are respectively given by $H^{I}_{\text {rem}}$ and $H^{O}_{\text {rem}}$ based on some vertex ordering ≺. They are defined as $H^{I}_{\text {rem}} = \{\;v\;|\;v\in V_{I} \wedge l(v)=\texttt {H} \wedge \exists _{\{v,v^{\ast }\}\in E_{I}} \wedge \exists _{v^{\prime }\neq v \in V_{I}} : (l(v')=\texttt {H} \wedge v^{\prime }\prec v \wedge \{v^{\prime },v^{\ast }\}\in E_{I})\;\}$ and $H^{O}_{\text {rem}}$ accordingly. Thus, any assignment *A* of *X*^*I*^ and *X*^*O*^ has to fulfill ${A^{I}_{i}}\not \in H^{I}_{\text {rem}}$ and ${A^{O}_{i}}\not \in H^{O}_{\text {rem}}$, which is implemented as a domain pruning preprocessing.

Furthermore, we can extend and tune the CSP formulation by comparing the graph structure of educts and products. To this end, we generate the multisets (denoted by 〈…〉) *N*_*I*_ and *N*_*O*_ of local neighborhoods of all atoms (vertices) for the educt and product graph, resp., given by

(1) $$\begin{array}{@{}rcl@{}}  N_{I} &=& \left\langle\; N(v) \;|\; v \in V_{I} \;\right\rangle \text{with} \end{array} $$

(2) $$\begin{array}{@{}rcl@{}}  N(v) &=& \left(\; l(v), \langle\; \mathcal{I}_{v,v^{\prime}} \oplus l(v^{\prime}) \;|\; \text{where}\right.\\  &&\left. v \neq v^{\prime} \in V_{I} \wedge \mathcal{I}_{v,v^{\prime}} > 0 \;\rangle\;\right) \end{array} $$

where *N*(*v*) is a tuple of the label of atom vertex *v* and an encoding of the multiset of all adjacent edges for this vertex. Note, ⊕ denotes string concatenation. *N*_*O*_ is derived accordingly. For example, the neighborhood multisets for the reaction from Figure 1 are

$$\begin{aligned} N_{I} &= \left\langle\; (\texttt{C},\langle1\texttt{C}\rangle), (\texttt{C}, \langle1\texttt{C},2\texttt{C}\rangle), (\texttt{C},\langle1\texttt{C},1\texttt{C},2\texttt{C}\rangle), \right. \\ & \left.\quad (\texttt{C}, \langle1\texttt{N},2\texttt{C}\rangle), 3\text{\(\times\)}(\texttt{C},\langle2\texttt{C}\rangle), (\texttt{N},\langle1\texttt{C}\rangle) \;\right\rangle \\ N_{O} &= \left\langle\; (\texttt{C},\langle1\texttt{C}\rangle), 3\text{\(\times\)}(\texttt{C},\langle1\texttt{C},1\texttt{C}\rangle), (\texttt{C},\langle1\texttt{C},1\texttt{C},1\texttt{N}\rangle), \right. \\ & \left.\quad (\texttt{C},\langle1\texttt{C},1\texttt{C},2\texttt{C}\rangle), (\texttt{C},\langle1\texttt{C},2\texttt{C}\rangle), (\texttt{N},\langle1\texttt{C}\rangle) \;\right\rangle \end{aligned} $$

Given the number of occurrences of an element *x* in a multiset *N*_∗_ by the multiplicity function ${occ}_{N_{\ast }}(x)$, the multiset subtraction *N*_*I*_∖*N*_*O*_ is defined by the occurrence reduction for each element *x*∈*N*_*I*_ to *max*(0,*occ*_*NI*_(*x*)−*occ*_*NO*_(*x*)). This subtraction *N*_*I*_∖*N*_*O*_ gives the local neighborhoods that are unique within the educts and thus are part of the ITS, i.e. have to be changed during the reaction. Therefore, we can derive a lower bound on the number of atoms of a certain type that are participating in the ITS. In the example this results in *N*_*I*_∖*N*_*O*_=〈 3 ×(C,〈2C〉),(C,〈1N,2C〉) 〉 revealing that at least 4 C-atoms of two neighborhood types (〈2C〉 and 〈1N,2C〉) are ITS members. The neighborhood types are educt/product specific, such that both *N*_*I*_∖*N*_*O*_ as well as *N*_*O*_∖*N*_*I*_ are computed.

**Figure 4:**
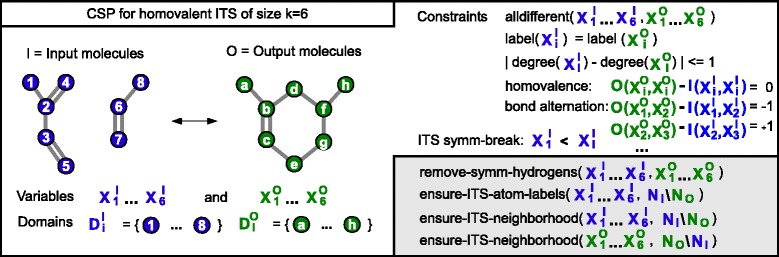
**Approach overview.** A simplified overview of the extended CSP for a homovalent ITS of size *k*=6 where the extensions of the basic CSP are given in the gray box in the lower right.

Given this information, we formulate an extended version of the basic CSP. Here, additional auxiliary node label variables $X^{L}=\left \{{X^{L}_{1}},\ldots,{X^{L}_{k}}\right \}$ are introduced, which encode the atom labels still possible for *X*^*I*^ assignments, i.e. ${D^{L}_{i}}=\left \{l(v)\;|\;v\in {D^{I}_{i}}\right \}$. Next, we derive the multiset of atom labels *N*^*L*^ to be present in the ITS with *N*^*L*^=〈*l*(*v*) | *N*(*v*)∈*N*_*I*_∖*N*_*O*_〉. In the example we find *N*^*L*^=〈C,C,C,C〉. To enforce the occurence of these atom labels in the ITS, we add for each each label *l* with ${occ}_{N}^{L}{l}>0\phantom {\dot {i}\!}$ an according global cardinality (count) constraint on *X*^*L*^. The basic atom label preservation constraint was extended to a ternary constraint that also propagates changes in *X*^*L*^ to both *X*^*I*^ and *X*^*O*^ and vice versa. In addition, we enforce that a valid assignment *A*^*I*^ of the ITS variables *X*^*I*^ reflects the explicit neighborhood *N*_*I*_∖*N*_*O*_, i.e., $N_{I}\setminus N_{O} \subseteq N(A^{I}) = \left \langle \;N\!\left ({A^{I}_{i}}\right)\;|\; 1\leq i\leq k\;\right \rangle $. An equivalent constraint is added for *X*^*O*^ to preserve the neighborhood *N*_*O*_∖*N*_*I*_, respectively. To minimize propagation cost, this is ensured by a simple n-ary constraint propagation after assignment. The CSP is illustrated in Figure 4.

Although the CSPs introduced above are defined for domains of vertices *v*∈*V*_*I*_∪*V*_*O*_, they can be easily reformulated using integer encodings of the atom indices allowing for the application of standard constraint solvers such as Gecode [35]. This enables the use of efficient propagators for most of the required constraints, such as the algorithm of Regin [36] for globally unique assignments. Only a few binary constraints, e.g. to ensure atom label preservation or the cyclic bond pattern, require a dedicated implementation as discussed in the Conclusions section.

All solutions for these CSPs are chemically valid ITS candidates. In order to check whether or not a true ITS is found we have to ensure that the remaining atoms, i.e., those that do not participate in the ITS, can be mapped without further bond formation or breaking. This is achieved using a standard graph matching approach as discussed in the following.

### Overall atom mapping computation

Given the CSP formulation from above, we can enumerate all valid ITS candidates. For a CSP solution we denote with ${a^{I}_{i}}$ and ${a^{O}_{i}}$ the assigned values of the variables ${X^{I}_{i}}$ and ${X^{O}_{i}}$, respectively. Once the ITS candidate is fixed, we can reduce the problem to a general graph isomorphism problem with a simple relabeling of the ITS edges. Thus, we derive two new adjacency matrices $\mathcal {I}^{\prime }$ and $\mathcal {O}^{\prime }$ from the original matrices  and , resp., as follows: For all atom pairs (*i*,*j*) within the cyclic index sequence 1-2-..-*k*-1, we change the corresponding adjacency information to a unique label using $\mathcal {I}^{\prime }_{{a^{I}_{i}},{a^{I}_{j}}} = \mathcal {O}^{\prime }_{{a^{O}_{i}},{a^{O}_{j}}} \in \{\, f,b\}$ encoding if a bond between the mapped ITS vertices is formed (*f*) or broken (*b*). All other adjacency entries are kept the same as in  and , respectively. In the following, we provide the ITS-bond-encoding adjacency matrices $\mathcal {I}^{\prime }$ and $\mathcal {O}^{\prime }$ for the example in Figure 1 given a 6-cycle ITS mapping (left) resulting from a CSP solution.



Bond formations within the ITS are encoded by *f* while bond breakings are encoded by *b*. These matrices in concert with atom label information are target to full graph isomorphism search to identify the complete atom maps. In the example only the atom mapping $m(v_{i})=v^{\prime }_{i}$ is found.

Given these updated “ITS encoding” adjacency matrices $\mathcal {I}^{\prime }$ and $\mathcal {O}^{\prime }$, the identification of the overall atom mapping *m* reduces to the graph isomorphism problem based on $\mathcal {I}^{\prime }$ and $\mathcal {O}^{\prime }$. Thus, all exact mappings of $\mathcal {I}^{\prime }$ onto $\mathcal {O}^{\prime }$ are valid atom mappings *m* of an elementary homovalent reaction, since the encoded ITS respects all constraints due to the CSP formulation.

### Extension to other ITS layouts

**Figure 5:**
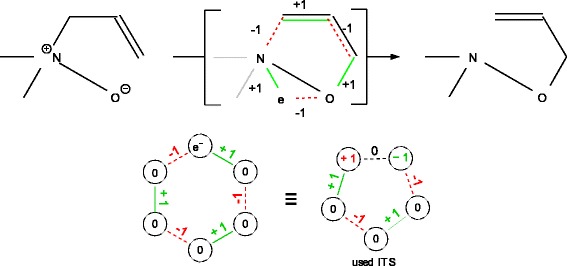
**Ambivalent reactions.** (top) The Meisenheimer rearrangement [37] transforms nitroxides to hydroxylamines. It does not admit a simple alternating cycle as ITS when molecules are represented as graphs whose vertices are atoms. An extended representation, in which the additional electron at the oxygen is treated as a “pseudo-atom” can fix this issue. (bottom) Note that such even sized cycles with a virtual vertex for the moving charge (vertex label *e* ^−^) can be represented by smaller odd cycles with two oppositely charged atoms separated by a non-changing pseudo bond (dashed edge labeled 0). See Figure 2 for further details of such an ITS layout.

Of course, not all chemical transformations are based on a homovalent elementary ITS. This will in general be the case for multi-step reactions and for the so-called ambivalent reactions, in which the number of non-bonding electron pairs (and thus the oxidation number of some atoms) changes in the course of a reaction [30]. Figure 5, for example, shows a reaction for which it is not possible to find a simple homovalent circular ITS using the presented ITS encoding. Still, the reaction shows a cyclic ITS with alternating bond electron changes for all but one bond [1].

We have extended the CSP-based framework outlined above to reactions with arbitrary cyclic ITS layouts, which allows for any defined bond and atom valence changes (i.e. charge changes) within the ITS. Figure 2 exemplifies odd ITS cycle layouts for ambivalent reactions [32]. The main difference to homovalent reaction CSP is the relaxation of the homovalence constraint, which is not enforced for all participating atoms [32]. Furthermore, the preservation of bond electrons for some ITS bonds instead of a change is enforced. The latter holds for instance for the bond connecting N^+^ and O^−^ in Figure 5.

### Implementation details

Our C++ implementation of the approach currently takes a chemical reaction in SMILES format [38], identifies chemically correct atom mappings, and returns these in annotated SMILES format. The latter provides a numbering of mapped atoms in the educts and products. It is available as C++ source code package v1.0.0 at http://www.bioinf.uni-freiburg.de/Software/.

Molecule parsing, writing, and graph representation uses the chemistry module of the Graph Grammar Library (GGL) [39]. We use an explicit hydrogen representation within the CSP formulation, as in [29], because most homovalent elementary reactions involve the replacement of at least one hydrogen. Unfortunately, the compact string encoding of molecules in SMILES format does not explicitly represent hydrogens. Thus, we use the hydrogen correction procedures of the GGL to complete educt and product molecule input. The CSP formulation and solving is performed within the Gecode framework on finite integer domains [35]. The final graph matching uses the state-of-the-art VF2-algorithm [40], which is among the fastest available [41].

The CSP uses standard binary order constraints and the *n*-ary distinct and counting constraints provided by the Gecode library. Dedicated binary constraints propagating on unassigned domains have been implemented for preservation of atom label, degree, and homovalence. The alternating cycle is implemented by a sequence of *k* constraints propagating the edge valence change of ±1. The ITS local neighborhood preservation to be enforced in the extended CSP is implemented by a dedicated n-ary constraint over all variables propagating on assignments only.

We are using a Depth-First-Search where the branching strategy chooses first variables with minimal domain size and first assigns non-hydrogen atom indices before hydrogen vertices are considered. The latter increases the performance to find the first solution since most reaction mechanism contain more than 50% non-hydrogen atoms. Once a non-hydrogen atom is selected for a variable, propagation will ensure that atom-adjacent hydrogens are considered for the variables adjacent within the ITS cycle encoding if appropriate.

For each ITS mapping identified, a full reaction atom mapping is derived via VF2-based graph matching. Therein, the discussed problem of hydrogen interchangeability (see Extended CSP formulation) is faced again and would result in symmetric overall atom mappings. This is countered by first producing intermediate “collapsed” educt/product graphs, where all adjacent non-ITS hydrogens are merged into the atom labels of their adjacent non-hydrogens. This preserves the adjacency information and enables a unique mapping via VF2 excluding the hydrogen-symmetries. Furthermore, this compression speeds up the graph isomorphism identification since the graph size is approximately halved.

While not described here, the CSPs can be easily extended to find candidates for the entire atom mapping by introducing additional matching variables for all atoms participating in the reaction, all constrained to preserve atom label, vertex degree, and bond valence information. But first tests (not shown) revealed that the increase in CSP size and accordingly search and propagation effort needed does not repay due to the efficiency of the VF2 graph isomorphism approach used. Therefore, we omitted this approach from this work.

## Application and evaluation

Benchmark sets for the evaluation of atom mapping methods are not readily available, since well-curated reaction databases, such as the KEGG REACTION database [42], do not provide detailed atom mapping information. Thus a manual data retrieval and curation was necessary to test the constraint-based atom mapping approach presented above.

### Predicting elementary reactions

The manual annotation of all of the about 10,000 reactions compiled in the KEGG REACTION database is infeasible with our resources. A data set comprising 630 manually curated atom maps for a subset of the KEGG database has been provided by [34]. Unfortunately, these atom mappings are restricted to non-hydrogen atoms. Thus they do not cover the whole reaction mechanisms, which usually involve hydrogen replacements. We therefore manually extended the data with the corresponding hydrogen mappings within the reaction center. Furthermore, the data set covers non-elementary reactions showing either multiple reaction centers or non-cyclic ITS. We found some atom mappings to be incorrect. We finally compiled a fully annotated data subset containing about 400 atom mappings of elementary reactions. The number of non-hydrogen atoms within the reactions ranges from 5 to 110 with a median of 36.

Studying the ITSs of these reactions, we found basically only 3 different ITS layouts covering 3–8 atoms. This exemplifies the very limited number of such layouts to be expected for elementary reactions. The ITS layouts found are visualized in Figure 2-top. Most reactions are homovalent (375) and only 14 are found to be ambivalent reactions that change atomic oxidation states. This shows the prominence of homovalent reactions.

We applied the prototypical implementation of our extended CSP formulation to the data set using the ITS layouts depicted in Figure 2. Runtimes were on average low with a median of 0.5 seconds. Nevertheless, there are about 20 reactions where atom mapping computations took longer than ten minutes. All of them are homovalent reactions of various ITS sizes. The increased runtime correlates with the number of involved atoms (Spearman rank correlation coefficient 0.79). Most such reactions contain large, connected static parts that cover about 90% of the involved molecules. Thus, we plan to incorporate an additional preprocessing to identify the small molecular subgraphs that are likely associated with the ITS and focus the CSP on these parts. This will result in drastically reduced search spaces and thus we can expect a substantial decrease of the running times. Atom mapping computations for ambivalent reactions were fast, which results from the additional constraints for the atomic oxidation state changes.

The resulting atom mappings were compared to the manually annotated data. We found only a single incorrect solution for a homovalent reaction according to the KEGG reaction mechanism classification (see Additional file 1): for R01440, our approach predicted an ITS of *k*=4, while the true mechanism involves *k*=6 atoms. Three reactions allowed for various mechanisms where the true atom mapping was contained in the set of alternative solutions predicted by our method. All atom mapping computations for ambivalent reactions were correct.

### Impact of the extended model

In order to investigate the impact of our extended CSP formulation over the basic version, we selected a representative subset of homovalent elementary reactions from the KEGG REACTION database. We restrict the evaluation to homovalent reactions due to the much higher computational cost. The latter emerges since we can not as easily identify ITS participating atoms as is the case for ambivalent reactions. The latter show at least one atom that changes its oxidation state, which confines the search space drastically.

The reactions have been chosen to provide various ITS and reaction sizes for evaluation. The average size of the selected reactions, i.e. the average number of atoms, is about 30 (Table 1 column 2) while the whole KEGG database shows an average of 50 atoms per reaction. The example reactions cover homovalent ITS sizes of *k*=4, 6, and 8 as introduced. Since there is no atom mapping information provided within the KEGG database, the example reactions had to be identified manually based on chemical knowledge. This again highlights the need for an automated identification of chemically feasible atom mappings as provided by our approach. The selected homovalent reactions are given in Table 2 with their respective KEGG ID, educts and products.

**Table 1:** **Performance evaluation of the basic and extended CSP model for reactions from Table** 2

				**Time**		**Sol.**	**Time all Sol.**	
**Reaction**	**Atoms**	**CSP Type**	***k***	**1st Sol.**	**Sol.**	**CSP**	**CSP**	**VF2**
R00013	14	Basic	6	0.03	1	346	0.8	0.03
		Ext. 〈2C〉		**0.02**		80	**0.05**	0.02
R00018	36	Basic	4	10.4	1	73,924	2.62	19.9
		Ext. 〈2N〉		0.28		36	0.44	0.01
R00048	30	Basic	4	0.1	2	26,178	1.44	6.1
		Ext. 〈2O〉		**0.02**		24	**0.42**	0.03
R00059	44	Basic	4	0.34	1	194,210	9.45	63.15
		Ext. 〈H,C,N,O〉		**0.03**		4	**2.08**	0.01
R00207	20	Basic	8	0.02	1	20,640	1.11	4.05
		Ext. 〈C,4O〉		**0.01**		24	**0.56**	0.02

Timings are given in seconds; minimal timings are highlighted in boldface. For extended CSPs, the minimal multiset of ITS participating atoms is listed in column 3. Column “Sol. CSP” gives the number of CSP solutions (ITS candidates) tested via VF2 for final atom mappings.

**Table 2:** **Elementary homovalent reactions from the KEGG REACTION database [** 42 **] used for the evaluation of the approach**

**Reaction**	**Educts**	**Products**
R00013	C(=O)=O, C(C(=O)O)(C=O)O	2×C(=O)(C=O)O
R00018	N, N(CCCCN)CCCCN	2×C(CCN)CN
R00048	CC(O)CC(=O)OC(C)CC(O)=O, O	2×CC(O)CC(O)=O
R00059	N(C(=O)CCCCCN)CCCCCC(=O)O, O	2×C(CC(=O)O)CCCN
R00207	P(=O)(O)(O)O, O=O, CC(=O)C(=O)O	P(=O)(OC(=O)C)(O)O, OO, C(=O)=O

The educt and product molecules are given in SMILES notation [38].

For each reaction, we applied our approach using both the basic and extended CSP formulation to evaluate the impact of the latter for various reaction and ITS cycle sizes. In Table 1 we report runtime, search, and solution details for the smallest ITS size *k* that yields a solution. For smaller values of *k*, the infeasibility tests were done within fractions of seconds and are therefore omitted.

Our atom mapping approach finds a first atom mapping for homovalent elementary reactions within milliseconds. It is clear that the additional constraints within the extended CSP formulation significantly increase the performance of the approach. This becomes even more striking when considering the timings for full solution enumeration. The extended CSP produces several orders of magnitude less ITS candidates (column “Sol. CSP”). Since the time consumption of the VF2 algorithm is about linear in the number of ITS candidates to test, this results in according speedups of the overall approach. Still there is room for optimization since the symmetry breaking within the CSP solution enumeration is not complete and ITS enumeration still allows for some symmetries (data not shown). The latter result from symmetries within the educt and product molecules, which are not handled by the simple ITS ordering applied so far. We are currently working on an extended generic symmetry identification and breaking for ITS, educts and products.

The strength of the extended CSP comes from the precomputed list of local neighborhoods to be part of the ITS candidate and the “hydrogen symmetry” breaking. For the reactions from Table 1, this list comprises on average about half the ITS resulting in the impressive impact of the constraint. For reaction R00059, the list covers the whole ITS with an according immense reduction in ITS candidates.

As already expected based on the results from other approaches [29], only a single or very few reaction mechanisms, i.e., non-symmetric atom mappings, are identifiable, see Table 2 column “Sol”.

## Conclusions

We have presented here the first constraint programming approach to identify chemically feasible atom mappings based on the identification of a cyclic imaginary transition state (ITS). The incorporation of the cyclic ITS structure within the search ensures the chemical correctness of the mapping that is not guaranteed by standard approaches that attempt to solve Maximum Common Edge Subgraph Problems [25]. To our knowledge, this is the first approach explicitly incorporating the cyclic ITS structure into an atom mapping procedure. The formulation of the CSP using only the atoms involved in the ITS results in a very small CSP that can be solved efficiently. Thus, it is well placed as a filter for ITS candidates for the subsequent, computationally more expensive graph matching approaches. The solutions of such an extended CSP are the desired chemically feasible atom mappings. We apply advanced symmetry breaking strategies and thus can enumerate all possible chemical mechanisms underlying a reaction.

The feasibility of the approach was introduced here for the common case of elementary, homovalent reactions, i.e., for reactions in which the transition state is an elementary cycle with an even number of atoms. We have shown that the CSP formulation can be easily extended to arbitrary cyclic ITS layouts. Usually, such reactions are not homovalent, i.e., at least one atom participating in the ITS is gaining or losing non-bonding electrons, which requires some moderate changes in the formulation of the constraints. We are currently identifying all feasible ITS layouts and are developing a generic CSP formulations for arbitrary layouts. This will result in a powerful approach to identify atom mappings with chemically valid ITSs.

At the moment, we apply a hierarchal combination of ITS-filtering via CP techniques followed by full atom mapping identification using a dedicated graph isomorphism algorithm. As already mentioned, there are also approaches to directly solve the graph isomorphism problem using CP [43-45]. While the used VF2 algorithm was shown to be efficient for first solution identification, other approaches (e.g. CP-based) show better performance for full solution space enumeration [46,47]. Currently, we are not aware of an available, efficient integration of the approaches in Gecode v4, such that they were not yet considered in this work. A prototypical implementation of graph isomorphism using the introduced constraints and propagators did not enable VF2-comparable runtimes (data not shown). Since we are dealing with molecular graphs of relatively simple structural complexity, the use of dedicated graph isomorphism algorithms, e.g. for planar graph [48], could increase the performance as well. Furthermore, other CSP encodings of the problem, e.g. by fusing current dedicated constraints like atom label preservation or homovalence into a single extensional table constraint [49], might improve the ITS identification step.

The current framework is designed to identify chemically feasible atom mappings for elementary, i.e. single-step, reactions. There are cases where short-lived intermediate molecules are formed that immediately react into the final products. Since these intermediate structures are unknown our present approach cannot be directly applied to such reactions. As noted by Hendriksen [2], often there is only a single unknown intermediate linking two consecutive elementary reactions. We therefore plan to create “fused” ITS layouts based on our single-step ITS encodings that will allow for the correct identification of atom mappings for multi-step reactions and reveal the individual steps and intermediate structures. For the combination of ITS layouts, we are currently investigating the multi-step reaction analyses by Fujita [50] and Herges [30].

Summarizing, we see constraint programming as a very promising approach to solve atom mapping problems since it provides a very flexible framework to incorporate combinatorial constraints determined by the underlying rules of chemical transformations.

## Additional file

Additional file 1
**Reaction mechanism comparison for reaction R01440, the only incorrect prediction.**
