# Supplementary material for: Atom mapping with constraint programming
Source: Algorithms Mol Biol. 2014 Nov 29;9:23. doi: 10.1186/s13015-014-0023-3 (PMC4256833; doi:10.1186/s13015-014-0023-3)
Supplement: Additional file 1 — Reaction mechanism comparison for reaction R01440, the only incorrect prediction. [file 13015_2014_23_MOESM1_ESM.pdf]

Atom Mapping with Constraint Programming

Supplementary Material

Martin Mann, Feras Nahar, Norah Schnorr, Rolf Backofen,
Peter F. Stadler and Christoph Flamm

S1 Reaction R01440

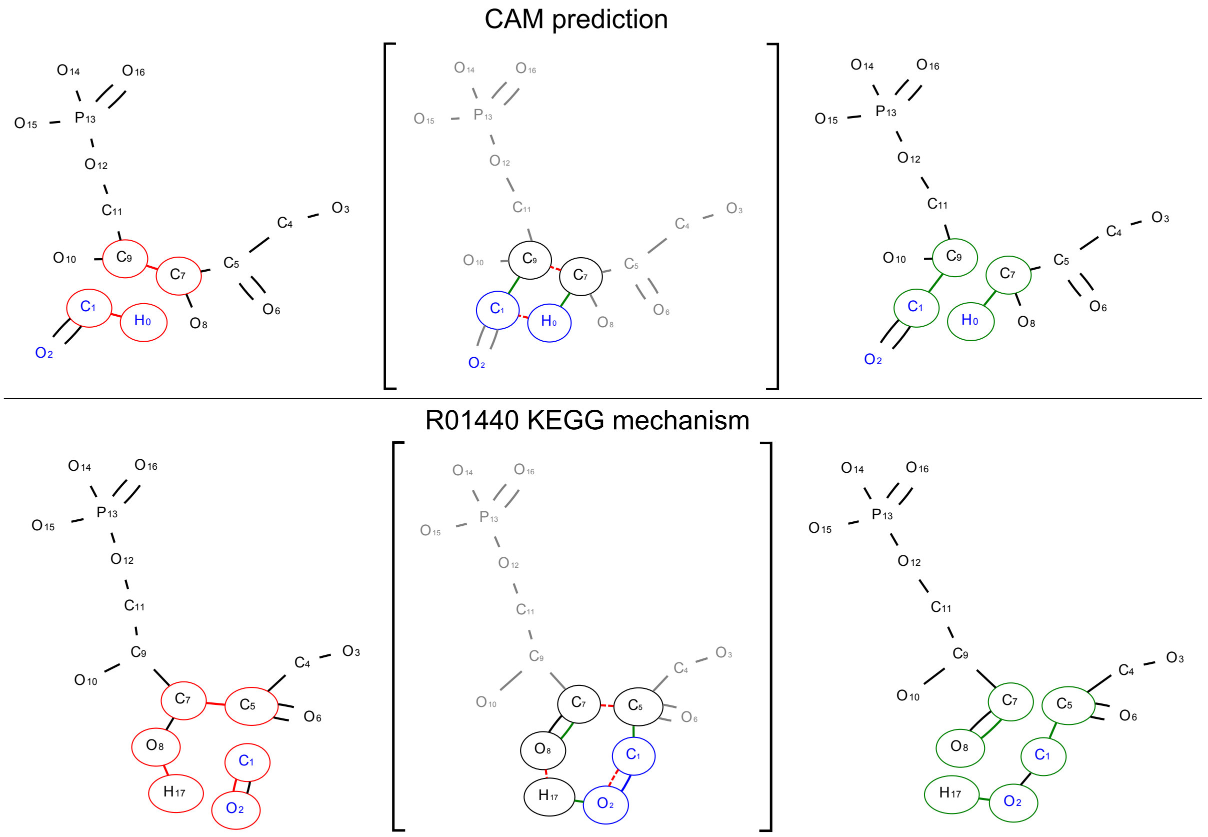

Figure S1: Reaction R01440 (top) CAM-predicted mechanism based on a homovalent ITS cycle of 4 atoms and (bottom) the 6-atom cycle according to the mechanism stored in the KEGG database. The center graph represents the ITS. Red bond are broken from left to right while green bonds are formed. Blue atoms belong to the `C=O` molecule from the left side.
